# Membrane nanotubes between peritoneal mesothelial cells: functional connectivity and crucial participation during inflammatory reactions

**DOI:** 10.3389/fphys.2014.00412

**Published:** 2014-10-24

**Authors:** Julia Ranzinger, Amin Rustom, Vedat Schwenger

**Affiliations:** ^1^Department of Nephrology, University of HeidelbergHeidelberg, Germany; ^2^Department of New Materials and Biosystems, Max Planck Institute for Intelligent SystemsStuttgart, Germany

**Keywords:** peritoneal dialysis, peritoneal mesothelial cells, inflammation, nanotubes, oxidative stress

## Abstract

Peritoneal dialysis (PD) has attained increased relevance as continuous renal replacement therapy over the past years. During this treatment, the peritoneum functions as dialysis membrane to eliminate diffusible waste products from the blood-stream. Success and efficacy of this treatment is dependent on the integrity of the peritoneal membrane. Chronic inflammatory conditions within the peritoneal cavity coincide with elevated levels of proinflammatory cytokines leading to the impairment of tissue integrity. High glucose concentrations and glucose metabolites in PD solutions contribute to structural and functional reorganization processes of the peritoneal membrane during long-term PD. The subsequent loss of ultrafiltration is causal for the treatment failure over time. It was shown that peritoneal mesothelial cells are functionally connected via Nanotubes (NTs) and that a correlation of NT-occurrence and defined pathophysiological conditions exists. Additionally, an important participation of NTs during inflammatory reactions was shown. Here, we will summarize recent developments of NT-related research and provide new insights into NT-mediated cellular interactions under physiological as well as pathophysiological conditions.

## Section

Peritoneal dialysis (PD) is an accepted alternative to hemodialysis in the treatment of end-stage renal disease. However, when compared internationally, PD-treatment is still underrepresented in Germany (Lameire and Van Biesen, 2010). In this treatment, the peritoneal membrane functions as semipermeable membrane allowing for ultrafiltration and diffusion (Krediet, 1999). In order to produce osmotically induced ultrafiltration, heat sterilized glucose containing dialysis solutions are used to ensure hyperosmolarity. The heat sterilization leads to the formation of glucose degradation products (GDPs) (Wieslander et al., 1995) which mainly contribute to the formation of advanced glycation end-products (AGEs) including methylglyoxal (MG) (Nakayama et al., 1997; Muller-Krebs et al., 2008). The implantation and presence of an indwelling catheter, high glucose concentrations, and GDPs in the dialysis solutions as well as peritonitis - a known complication of PD - coincide with high levels of proinflammatory cytokines within the peritoneal cavity and subsequent induce chronic inflammatory conditions leading to structural and functional changes of the peritoneal membrane (Witowski et al., 2000; Flessner et al., 2007, 2010).

Moreover, a strong induction of the expression of the receptor for advanced glycation end-products (RAGE) in the peritoneal membrane of uremic patients was demonstrated which further increased after PD treatment (Kihm et al., 2008), showing AGE-RAGE interactions being crucial in peritoneal damage due to inflammatory conditions, uremia, and PD. The resulting loss of ultrafiltration, as well as progressive fibrosis, angiogenesis, and vascular degeneration limit long-term PD-treatment (Margetts and Churchill, 2002; Devuyst et al., 2010).

It is now clear that peritoneal mesothelial cells, specialized epithelial cells that line the peritoneal cavity, not only function as non-adhesive surface to facilitate intracoelomic movement. These cells are crucial for the maintenance of peritoneal homeostasis, transport processes across the peritoneal membrane and tissue repair (Mutsaers, 2004; Yung and Chan, 2007). Furthermore, they provide defense against bacterial insult and are essentially exposed to the bioincompatible dialysis solutions during PD-treatment. In response to Tumor-Necrosis-Factor (TNF) and Interleukin-1 (IL-1) secreted by peritoneal macrophages, peritoneal mesothelial cells synthesize various cytokines, including IL-1, IL-6, and IL-8, thus enhance the inflammatory signal and recruit leukocytes in the peritoneal cavity (Douvdevani et al., 1994; Topley, 1995; Li et al., 1998). In the immune system, peritoneal mesothelial cells bear an effective antigen-presenting function for T cells and thereby play a relevant role during the immune response in the peritoneal cavity e.g., during peritonitis (Valle et al., 1995; Hausmann et al., 2000). However, with increasing PD-treatment, peritoneal mesothelial cells undergo a progressive loss of their epithelial phenotype toward a myofibroblast-like phenotype (Yanez-Mo et al., 2003). Thereby, a loss of characteristic cell-cell junctions, apical-basal polarity as well as reorganization of the cytoskeleton and reprogramming of the gene expression take place (Lamouille et al., 2014). This differentiation process, known as epithelial-mesenchymal transition (EMT), reflects the enormous plasticity of mesothelial cells (Yung and Chan, 2012).

**Figure 1:**
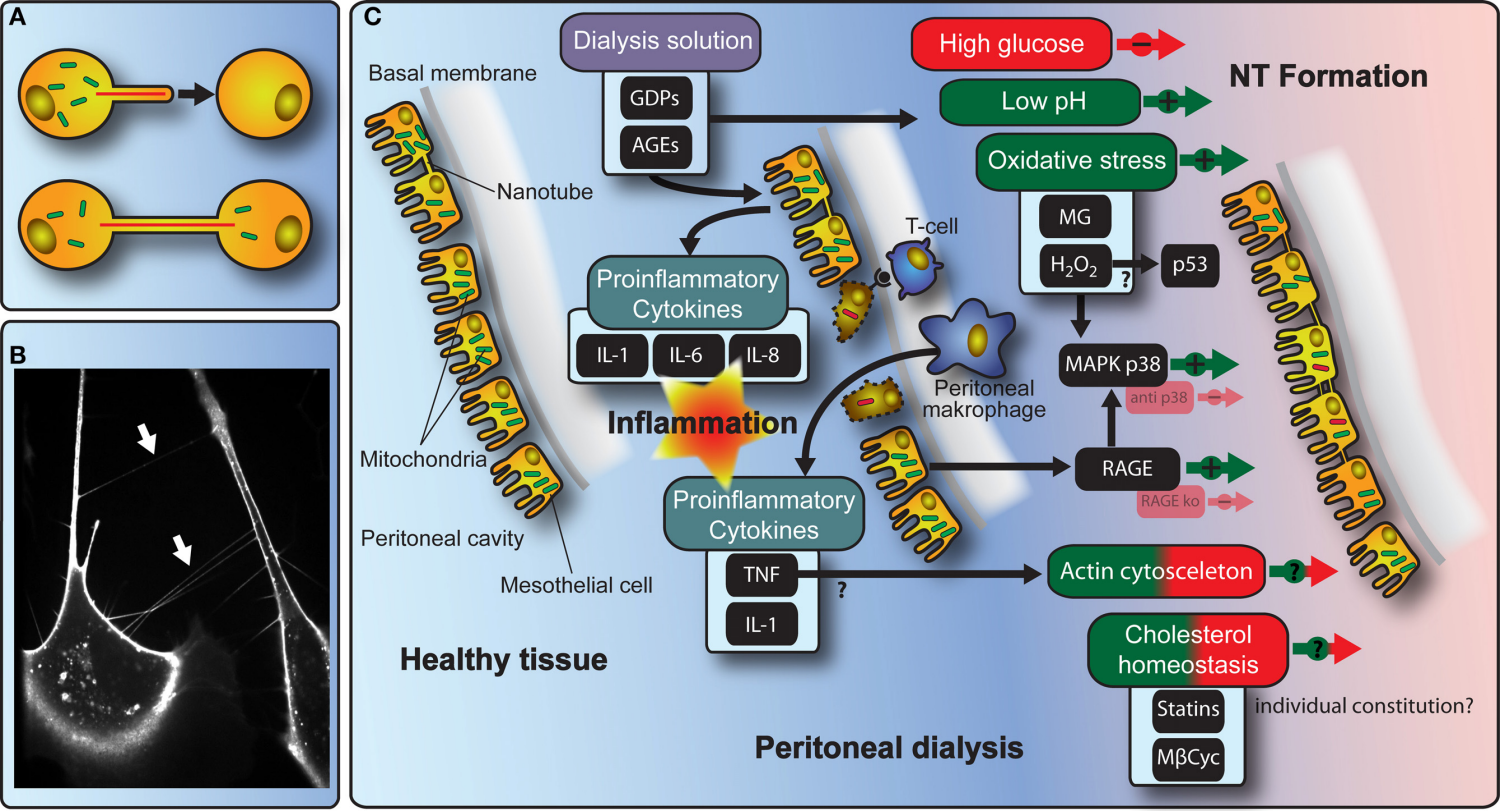
**Schematic model of molecular mechanisms with implications for NT-mediated cellular interactions during PD. (A)** The cartoon depicts the principle of NT-formation and NT-mediated transfer of organelles between cells. **(B)** The fluorescence microscopy picture shows NTs (arrows) spanned between primary human peritoneal mesothelial cells. **(C)** Illustration of the interdependence of NT-formation and communication in the context of PD-treatment including stimulating/inhibiting factors.

In the context of inflammatory immune reactions, intercellular communication plays a crucial role. In 2004, the discovery of Nanotubes (NTs) mediating membrane continuity has extended the understanding of cell-to-cell communication (Rustom et al., 2004). These NTs were initially characterized as thin intercellular membrane channels, formed between cultivated pheochromocytoma (PC12) cells at their nearest distance and without contact to the substratum (Figure 1A), displaying diameters from 50 to 200 nm and lengths of up to several cell diameters (Rustom et al., 2004). NTs contain F-actin and/or microtubule backbones and facilitate the intercellular transmission of various cellular components, including organelles as well as plasma membrane constituents or the transfer of electric signals (Hurtig et al., 2010; Wang et al., 2010; Wang and Gerdes, 2012). Meanwhile, NTs have been found to be present in cultures of different cell types including for example mesothelial (Figure 1B) and epithelial cells, fibroblasts, immune cells, and neurons (Vidulescu et al., 2004; Castro et al., 2005; Watkins and Salter, 2005; Davis and Sowinski, 2008; Gerdes and Carvalho, 2008; Pontes et al., 2008; Ranzinger et al., 2011). Moreover, recent research demonstrates the existence of NTs in human primary tumors (Lou et al., 2012).

During the last 10 years, a lot of knowledge concerning formation, function, and biological implications of NTs in health and disease has been gained. Aside from mediating functional connectivity between various cell types, it is now clear that NTs participate in several pathological processes of substantial medical interest. NTs were proposed to be involved in the intercellular spread of prion proteins (Gousset and Zurzolo, 2009; Dunning et al., 2012) and viral proteins, e.g., during HIV infections (Eugenin et al., 2009; Kadiu and Gendelman, 2011; Sowinski et al., 2011), the transfer of drug resistance between cancer cells (Pasquier et al., 2012) or the transfer of Aβ peptides in the context of Alzheimer's disease (Wang et al., 2011). Moreover, it could be demonstrated that the number of NTs in humans is dependent on the individual donor background (Ranzinger et al., 2011) and correlates with defined pathophysiological conditions. In *in vitro* experiments, in which cells were stimulated with TNF, the number of NTs is significantly increased and associated with a remodeling of the actin cytoskeleton. This finding could be confirmed when NT-numbers were investigated between cells from PD-patients (Ranzinger et al., 2011) pointing to an important participation of NTs during inflammatory reactions.

Additionally, a strong correlation of NT occurrence with cellular cholesterol contents and its distribution throughout the cell could be demonstrated. Experiments, in which cellular cholesterol homeostasis in HPMCs was affected by cholesterol depletion via methyl-β-cyclodextrin (MβCyc), revealed that gradual cholesterol depletion results in a strong, non-linear modulation of NT-numbers and lengths with significant peaks at given MβCyc concentrations, pointing to narrow windows of defined cholesterol contents being beneficial or detrimental, e.g., by affecting NT tensile strength or by influencing the formation process (Ranzinger et al., 2011). The finding that statin-treatment of HPMCs resulted in significantly increased NT-numbers coincides with comparably high numbers of NTs found between cells from a patient undergoing statin treatment (Ranzinger et al., 2013). In a recent study, Thayanithy et al. (2014) explored exosomes and lipid rafts as mediators of NT-formation in mesothelioma cells. Their results provide evidence for exosomes as chemotactic stimuli for NT-formation and lipid raft formation as potential biomarker for NT-forming cells.

To date, aside from inflammatory conditions, several factors are known that lead to the induction of NT-formation, among these oxidative stress as well as several receptor-ligand interactions (Martinez et al., 2002; Zhu et al., 2005; Chinnery et al., 2008; Ranzinger et al., 2011; Wang et al., 2011; Sun et al., 2012). In the context of PD-treatment, the use of dialysis solutions lead to a significant reduction in NT-numbers between peritoneal mesothelial cells (Ranzinger et al., 2011). Observed more closely, oxidative stress caused by both methyglyoxal (MG) and acidified pH-value results in higher NT-numbers whereas alterations in cellular osmolarity due to enhanced glucose concentrations lead to a strong decrease in NT-numbers between the cells (Ranzinger et al., 2014). In this context, by blocking of RAGE, whose expression is upregulated during PD-treatment, it could be shown that this receptor is a strong regulator in NT-formation processes between murine and human peritoneal mesothelial cells *in vitro* and *in vivo* (Ranzinger et al., 2014).

Respective underlying molecular mechanisms involved in the formation of NTs are controversially discussed. Studies from Wang et al. (2011) showed that NT-formation in primary rat hippocampal astrocytes and neurons is dependent on the activation of the tumor suppressor protein p53 through hydrogen peroxide induced cellular stress. Andresen et al. (2013) however showed that p53 is dispensable for NT-formation in SAOS-2 cells and dKO-MSCs. The results of these studies demonstrate that signaling pathways and involved proteins having an impact on the formation of NTs act strongly cell-type dependent. A previous study from Zhu et al. (2005) showed that oxidative stress induced by H_2_O_2_ increases the formation of NTs in astrocytes through activation of the p38 mitogen-activated protein kinase (MAPK) pathway. In the context of RAGE being involved in the formation of NTs between peritoneal mesothelial cells, the MAPK signaling cascade, which is addressed upon RAGE activation, was investigated in a recent study from Ranzinger et al. (2014). It could be demonstrated that oxidative stress induced by MG not only induces NT-formation but also increases phosphorylated p38 protein levels. Subsequently, blocking of p38 resulted in reduced NT-numbers between the cells arguing that the action of p38 regulates NT-formation in peritoneal mesothelial cells.

The existence of NTs *in vivo* has been supported by an increasing number of publications (Eugenin et al., 2009; Pyrgaki et al., 2010; Caneparo et al., 2011; Ranzinger et al., 2014). However, their occurrence, architecture, and function in the body is still a matter of considerable debate and may vary in accordance to the respective species, tissue, developmental stage, age, genetic background, and pathophysiological variations. One possible function could be a NT-mediated rescue for cells and/or organs under pathophysiological conditions like oxidative stress (Figure 1C).

In this view, several studies showed e.g., the transfer of mitochondria via NTs (Koyanagi et al., 2005; Domhan et al., 2011; Wang et al., 2011). A study from Vallabhaneni et al. (2012) showed that vascular smooth muscle cells initiate proliferation of mesenchymal stem cells through the exchange of mitochondria in co-cultures. Pasquier et al. (2013) demonstrated that cancer cells acquiring mitochondria from endothelial cells display significant chemoresistance. Furthermore, work from Spees et al. (2006) could demonstrate that aerobic respiration in cells with dysfunctional mitochondria could be rescued by mitochondrial transfer. More recently, concerning ischemia/reperfusion (I/R)-induced injury of the kidney, in a RAGE knockout mouse model, it could be shown that under conditions where RAGE is absent, NT-numbers are increased and kidney tissue morphology is improved compared to kidneys from wild-type mice (Ranzinger et al., 2014). Interestingly, when RAGE is blocked and dialysis solution as secondary stimulus has been applied, increased NT-numbers could also be demonstrated in peritoneal mesothelial cells (Ranzinger et al., 2014). In these cases, one could speculate that a protective effect for the respective organ/tissue might be attributed to an increased NT-formation accompanied by frequent exchanges of for example mitochondria.

In the future, further investigations are needed to investigate NT-mediated transport mechanisms within the peritoneal cavity in greater detail. This will have significant impact on the understanding of a variety of processes, such as inflammatory immune reactions. Potentially, this knowledge will allow for the development of improved treatment options during peritoneal infection.

### Conflict of interest statement

The authors declare that the research was conducted in the absence of any commercial or financial relationships that could be construed as a potential conflict of interest.
